# Improving the Efficiency of 3D Monocular Object Detection and Tracking for Road and Railway Smart Mobility

**DOI:** 10.3390/s23063197

**Published:** 2023-03-16

**Authors:** Alexandre Evain, Antoine Mauri, François Garnier, Messmer Kounouho, Redouane Khemmar, Madjid Haddad, Rémi Boutteau, Sébastien Breteche, Sofiane Ahmedali

**Affiliations:** 1 Univ Rouen Normandie, Normandie Univ, ESIGELEC, IRSEEM, 76000 Rouen, France; 2 SEGULA Technologies, 19 Rue d’Arras, 92000 Nanterre, France; 3 Univ Rouen Normandie, INSA Rouen Normandie, Université Le Havre Normandie, Normandie Univ, LITIS UR 4108, 76000 Rouen, France; 4 IBISC, Evry-Val-d’Essonne University, Universite Paris-Saclay, 91080 Évry-Courcouronnes, France

**Keywords:** monocular 3D object detection, dataset combination, knowledge distillation, 3D bounding boxes estimation, object localization, distance estimation, 3D multi-object detection, deep learning, smart mobility

## Abstract

Three-dimensional (3D) real-time object detection and tracking is an important task in the case of autonomous vehicles and road and railway smart mobility, in order to allow them to analyze their environment for navigation and obstacle avoidance purposes. In this paper, we improve the efficiency of 3D monocular object detection by using dataset combination and knowledge distillation, and by creating a lightweight model. Firstly, we combine real and synthetic datasets to increase the diversity and richness of the training data. Then, we use knowledge distillation to transfer the knowledge from a large, pre-trained model to a smaller, lightweight model. Finally, we create a lightweight model by selecting the combinations of width, depth & resolution in order to reach a target complexity and computation time. Our experiments showed that using each method improves either the accuracy or the efficiency of our model with no significant drawbacks. Using all these approaches is especially useful for resource-constrained environments, such as self-driving cars and railway systems.

## 1. Introduction

Autonomous vehicles are increasingly present in our daily lives, opening up new perspectives in mobility and transportation. These vehicles evolve in a dynamic environment shared by many other users. The R&D work of our team is directly related to one of the major issues of this field, which is the perception of the environment for autonomous vehicles, namely autonomous cars, autonomous trams, and autonomous trains. In the context of intelligent mobility applications, object detection and accurate depth estimation are necessary for safe navigation. A good perception of the environment requires understanding the following issues:

- Mapping and localization: this represents the establishment of spatial relationships between the vehicle and static surrounding objects.
- Detection and tracking of moving objects
- Object classification (car, bus, pedestrian, cyclist…): The vehicles need to be able to detect each different class precisely, as each class has its own behavior, and properly identifying each object is key to understanding the environment.

In the scope of our work, only the two latter issues are relevant. To solve these, Deep learning algorithms allow the computer to analyze the shapes and objects that make up an image, following two main methods:

- Classification and localization algorithms draw bounding boxes around each object detected.
- Instance segmentation associates each pixel with a class to uniquely identify it. The segmentation trims the object and is more accurate than the bounding box detection.

Among all these approaches, in our case of autonomous cars and trains, 3-dimensional object classification is the most relevant method.

In addition to this, additional challenges have been identified. The first one is the availability of databases in the railway and road domains, rich enough (both in terms of the number of images and data as ground truth) to allow the adequate training of deep learning methods. While there are plenty of datasets covering the road domain, datasets covering the railway domain for object detection are much less numerous. As our work used 3D detection, what we needed was datasets with not only ground truth data about the objects’ classes and positions on the pictures, but also their real location, dimensions, and orientations.

The second challenge identified concerns the design of a solution for real-time detection, localization, and trajectory prediction in a complex and varied environment compatible with limited onboard processing resources. The optimization solutions have to combine the detection performance without altering the speed of the inference model. Embedded systems present a major internal limitation for our work, this being the limitation of computing power, while artificial intelligence algorithms present an extreme computing costs. We, therefore, overcame this limitation by using an architecture capable of providing a high level of parallel processing, also called embedded GPU, namely, NVIDIA Jetson TX2. We tried to find the balance between the performance of the neural network, i.e., its ability to perceive its environment, and the execution speed inherent to the embedded GPU. Our aim was to provide an answer to both challenges by making full use of dataset combination, and by developing and improving a monocular 3D object detection and tracking system that can provide real-time performance, even on embedded systems, with limited processing resources.

This paper is organized as follows: Section 1 introduces this paper. In Section 2, we review the related works, YOLO-based object-detection algorithms, existing datasets for smart mobility, methods to extract synthetic data, object tracking, and, finally, knowledge distillation. In Section 3, we introduce the work done by our team in regard to our improvements on YOLOv5-3d [1] using tracking visualization, and dataset combination. The implementation of our algorithm on an embedded system for field usage is described in Section 4. Experimental results, whether qualitative or quantitative, are the subject of Section 5, where we show the results of the dataset combination, the knowledge distillation, and the development of the lightweight model. Finally, the conclusions and future directions are outlined in Section 6.

## 2. Related Works

### 2.1. Object Detection Methods

The best-performing Deep Learning-based methods for object detection are generally based on a convolutional network separated into two modules. The first one is dedicated to the region proposal, which returns the coordinates of the bounding boxes in which an object is possibly present. The second module then performs the detection and returns the class of the object present in the proposed region. Although the detection performance of these methods is excellent, they are computationally intensive and not well suited for real-time applications. Instead, the YOLO [2] algorithm proposes a different approach and uses a single convolutional network to predict the position of objects (region) and their class, while keeping consistent detection performance. It is for this reason that we chose to focus on YOLO, rather than on other multi-stage object detection methods. The different versions of YOLO [3,4,5] have brought many improvements to the original version. Training using batch normalization pushes each layer of the network to have more or less the same distribution at each step of the training and, thus, gains in accuracy. Replacement of the fully connected layers, responsible for the detection, by “anchor boxes”, similar to the default boxes of SSD, is another improvement. In addition, the network responsible for extracting the image features has been replaced by the Darknet network, which has 53 convolutional layers instead of 19. These new methods, therefore, offer better detection performance than the original YOLO, while being inexpensive in terms of computing time.

In terms of its operation, YOLO divides the input image into a set of grids of varying fineness. It then applies convolutions and predicts whether an object is located in a zone of the grid. However, it is the agglomeration of these “anchor boxes” that allows us to make the final detection of the object by applying the NMS function, or removal of non-maximum anchor boxes. It is this cutting in the form of a grid that allows YOLO to detect both small and large objects in the image, while gaining in speed compared to other detection methods. The version of YOLO we used [1] was a 3D monocular detection method built on YOLOv5 [5], which replaces YOLOv5’s original anchor boxes (containing class information as well as location information within the picture) with hybrid anchor boxes, which contain more information, such as the following: distance from the camera, 3D centers projected on the image plane as well as 3D dimensions and orientation. All these pieces of information together allow us to precisely locate an object’s position and orientation in a 3D environment. We made further improvements to this method, which aim to provide monocular 3D object detection with the main focus being real-time performance. While there are some similar methods, such as in [6,7,8], these methods either require stereo or depth cameras or are more focused on precision, rather than real-time performance. In addition, during, or soon before, our work, other versions of YOLO were released, such as [9,10,11,12]. Among these, the most interesting one is [12], which offers gains in both time and performance without any drawbacks. While using it has not been a possibility during our work so far, it is our intent to port our adaptation of YOLOv5 to YOLOv7 in order to benefit from the latter’s considerable improvements.

While we focused mostly on YOLO-based approaches, since these were methods similar to ours, there are many object detection algorithms with their own unique approaches. Among these, [13,14,15] are some of the most recent monocular 3D object detection methods, while [16,17] are the 2021 SOTA in this domain. Among these monocular 3D object detection approaches [18,19], are methods specifically aimed on achieving high real-time capabilities like ours. However, the former was tested on two RTX 1080 GPUs, while the latter used 2 NVIDIA Tesla V100 GPUs. This differs from our approach, which mostly utilized embedded systems, like the NVIDIA Jetson TX2, for its real-time performance evaluations, as shown in Table 1.

### 2.2. Datasets

The volume and quality of data in a learning problem remain a fundamental issue, as well as the structure of the model to be trained. The field of autonomous cars, being in full development, has a multitude of data sets for the road domain, such as KITTI [20], which provides images from a stereoscopic camera, with the depth of the scene measured by a Velodyne LIDAR, as well as vehicle and pedestrian annotations for object detection, or NuScenes [21], which has six cameras and a LIDAR with a large volume of data that can be used for 2D and 3D detection and tracking. There are also other datasets available, like CityScapes [22], Pascal VOC [23], ImageNet [24] or MS-COCO [25], but they were not used during our work because they did not contain the information we needed. We used KITTI and NuScenes, mainly because they are both open-source datasets dedicated to 3D detection for autonomous vehicles and both contain sequences of videos from urban environments. These datasets were especially useful for this project because they feature most of the situations which might be encountered during real-life use of the algorithms.

In addition, there are simulators that allow automatic acquisition and annotation, such as in CARLA [26] or SYNTHIA [27]. However, the graphical rendering of simulators is not always up to par with reality, due to rendering quality, texture issues, dated graphics, and domain shifts, and were not photorealistic enough for our methods once applied in real conditions. An acquisition from Grand Theft Auto V [28] (GTA V), a video game with a photorealistic appearance, allowed us to obtain a large virtual dataset for 3D detection capable of being combined with our previous datasets. There are several articles explaining how to acquire data from this specific game, such as Richter’s [28] works, which include instructions and available code to use, and Philipp Krähenbühl’s Gamehook [29], a tool allowing extraction of the rendering code of a video game.

In addition to the hybrid datasets used during these works, our team also developed a rail–road real–synthetic hybrid dataset called ESRORAD [30]. To develop the hybrid road–railway dataset, we used a combination of synthetic data generated using GTA, as well as real image recordings. We used GTA to generate a large number of images of roads and railways, along with their corresponding labels and ground truth data. To augment the synthetic data with real examples, we also collected a set of real images of roads and railways by using a car equipped with captors and driving over an existing railway. We then manually annotated a subset of the images (approximately 2500) for use in our algorithms, resulting in a hybrid dataset that includes both synthetic and real-life data.

For the railway domain, the number of available datasets is much lower: we have so far only identified two datasets, RailSem19 [31], and FRSign [32]. Railsem19 offers 8500 images for semantic segmentation, FRSign has 100,000 images for railway traffic light recognition, but both of these datasets are not relevant in the case of 3D semantic segmentation. The ESRORAD dataset is, as of now, the only dataset offering both road and railway data freely available with ground truths, making it a valuable resource for researchers and developers working on object detection and classification in the railway field.

### 2.3. Object Tracking

In the context of autonomous cars, knowing the position in space of the objects surrounding the vehicle is not sufficient, since they are usually in motion. For this reason, we used object tracking to anticipate their dynamic behavior and predict dangerous situations. Object Tracking allowed us to make the detection association, that is to say, to recognize a determined object between two images and to follow it in time. These tools use sequences of images and, thus, make an extraction both spatial and temporal (distance, velocity, texture, etc.). Although very useful, these methods do have limitations:

- Occlusion and truncation, when an object is totally or partially hidden by another, or when it goes out of the camera’s field of view, cause the algorithm to forget the object, and once it is back in the frame, the algorithm considers it to be a new object.
- Fast camera or point of view movements can cause the algorithm to predict erroneous trajectories, as it attributes these sudden important changes in the frame to the objects’ movements.

Nevertheless, we consider that, in the context of a fixed camera on a vehicle following a smooth trajectory, these problems do not prevent the tracking methods fulfilling their obstacle avoidance function.

There are several methods of MOT (Multiple-Object Tracking) available, some based on the use of Kalman filters, such as SORT [33] (Simple Online and Real-time Tracking), while others use CNNs (Convolutional Neural Networks), like DeepSORT [34] or Trackformer [35]. The advantage of Kalman-based solutions is that they are able to work directly, by using the output of a separated object detection algorithm, to initiate the tracking and improve it during the sequence. On the other hand, Tracking solutions using Deep Learning often perform both object detection and tracking, making them partially redundant with our already existing object detection algorithm. Since real-time performance was an important issue for us, we could not afford to perform two object detection in parallel, as it would be a tremendous waste of computing resources.

### 2.4. Knowledge Distillation

Knowledge distillation, as defined in [36], is a neural network optimization method. Indeed, artificial intelligence models require enormous means in terms of computing power to be trained. Moreover, in most cases, these models are implemented in systems with little power. Knowledge distillation allows the overcoming of this problem of model minimization for embedded systems, smartphones, etc.

It consists of training two models in parallel: a teacher network and a student. The teacher is a network already trained with large and complex layers, allowing it to obtain high accuracy scores (in mAP, position accuracy, and dimensions accuracy). The student is a light network, with thinner and less deep layers, allowing it to have a higher speed of execution compared to the teacher model. A survey on knowledge distillation [37], outlined the three main types of distillation schemes (offline distillation, online distillation, and self-distillation), as well as some of the latest distillation algorithms, such as [38,39].

## 3. Improvements on 3D Object Detection and Tracking

### 3.1. Object 3D Detection & Classification: Improvements on YOLOv5-3D [1]

To create a 3D object detection algorithm from YOLOv5, we modified the network architecture to add additional outputs for 3D information. This involved adding new layers to the network that could predict the 3D center of the object, as well as its dimensions and orientation, based on the 2D bounding box predicted by YOLOv5. Next, we trained the YOLOv5-3d [1] network on a dataset of images with corresponding 3D ground-truth labels. The resulting 3D object detection algorithm was able to accurately predict not only the class and 2D bounding box of each object, but also its 3D center, dimensions, and orientation. While this algorithm has already been the subject of its own article [1], we made some improvements such as improved hyperparameters (results shown in Table 2), as well as the use of new methods, namely the integration and modification of the SORT [33] algorithm for tracking, the use of dataset combination, knowledge distillation and the creation of a lightweight model (i.e., Table 1).

One of the advantages of our 3D detection algorithm is that it has a single-stage architecture and a limited number of layers, which makes it highly efficient and allows it to achieve good real-time performance. Our algorithm can be run on a wide range of devices, including embedded systems with low computational capacities, such as the Jetson TX2. In the field of autonomous vehicles, it is critical that the algorithms used for object detection and localization can operate in real-time, because the decisions made by these algorithms can have significant consequences, such as avoiding collisions with other objects. Table 2 shows the quantitative evaluation of YOLOv5 on the KITTI dataset.

One of the main limitations of YOLOv5-3d is that it is extremely dependent on the resolution of the training images. If the images used during inference have a different resolution than the images during training, this negatively affects the accuracy of the different predictions, especially the distance prediction, as seen in Table 2. In this table, we can see that the RE (Relative Error) and RMSE (Root Mean Square Error) are both higher after a change in resolution between training and inference.

Additionally, the accuracy of our algorithm is also dependent on the camera’s intrinsic parameter differences between the training and inference. These parameters include the focal length of the camera, the size of the pixels on the camera’s sensor, and other factors that affect the way the camera captures images. If the intrinsic parameters of the camera used for inference are different from those used during training, the accuracy of the algorithm may be reduced.

It is possible to easily mitigate these limitations of YOLOv5-3d by changing the dimensions of the input images, with both resizing and cropping, as well as by using OpenCV [40]’s undistort function, that reprojects an image from one camera matrix to another.

The only downside of this solution is that it requires the picture to be pre-processed, which makes this fix hard to apply in embedded systems, which is why, rather than trying to adjust the inference images to fit the training images’ resolutions and camera matrices, the best solution is to instead pre-process the training data. Once the model is trained with pre-processed data already fitting the inference’s resolution and camera matrix, the inference works as intended without requiring any additional pre-processing.

In order to improve our model, we decided to create a lightweight version specifically for use in embedded systems, such as the Jetson TX2. The goal of this model is to have improved real-time performance and to run quickly, even on systems with limited resources. We used the Jetson TX2 as a reference during our development, as it is a powerful embedded system that is commonly used in a variety of applications. The NVIDIA (Santa Clara, CA, USA) Jetson TX2 features a 256-core NVIDIA Pascal^TM^ GPU architecture with 256 NVIDIA CUDA cores, a Dual-Core NVIDIA Denver 2 64-Bit CPU and a Quad-Core ARM Cortex-A57 MPCore, 8GB of 128-bit LPDDR4 memory running at 1866 MHz, and 59.7 GB/s of memory bandwidth. These specifications make the Jetson TX2 a good reference platform for our lightweight model, as it is an embedded system that provides a balance of computational power and energy efficiency. By designing the model to work well on the Jetson TX2, we could ensure that it would perform well on a wide range of embedded systems with similar specifications, rather than being limited to only high-end systems.

A network that has too many parameters has a much higher computation time while having a greater tendency to overtrain, which degrades its accuracy considerably. On the other hand, a model that does not have enough parameters does not learn well and has lower accuracy. In our network, the number of parameters defining its complexity depends largely on the number of layers and filters. The speed and accuracy of our approach also depends on the resolution of the input images. Work has already been done to obtain an optimal set (depth, width, image resolution) in EfficientNet [41,42]. The authors determined that the complexity of the model, measured in Flops, was related to its width (*w*) (#layers), depth (*d*) (#channels), and resolution of the image (*r*), as described in Equation (1):

(1) $$Flops∼w^{2}\times r^{2}\times d$$

In order to create an even lighter model, we chose to take a similar approach. First, we defined the complexity of our model with the objective of obtaining a computation time of 33 ms/image on the Jetson TX2. The chosen complexity was approximately 10 GFlops. Starting from an initial complexity of 550 GFlops, we then selected all the combinations (width, depth, resolution) that would allow us to obtain the target complexity. Finally, we trained each of these models for 20 epochs on GTA. If we define w=1 and d=1 as the width and height of the “Large” model and r=1 to define an image of resolution 1280×720, the model presenting the best balance between performance gain and precision we found had the combination (w=0.4, d=0.5, $r=\frac{576}{1280}=0.45$). Other w/d/r combinations tested empirically had either too much of a precision decrease or not enough performance gain when compared to this one. We later adopted the same procedure to train the other models (Large, Medium, etc.).

### 3.2. Object Tracking & Visualization

**Figure 1:**
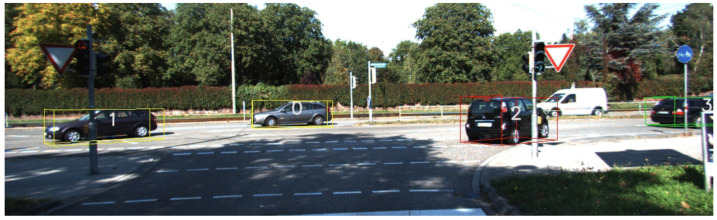
Object tracking with 3D bounding boxes and tracking numbers.

In the context of autonomous cars, knowing the position in space of the objects surrounding the vehicle is not enough, since other vehicles are usually in motion and collisions must be avoided. Therefore, in order to anticipate their behavior and the trajectories to avoid dangerous situations, we used multiple-object tracking solutions. We integrated and modified the SORT [33] algorithm for this purpose. SORT only works for 2D detection, and we modified its Kalman algorithm to move from 2D to 3D. The results can be seen in Figure 1.

**Figure 2:**
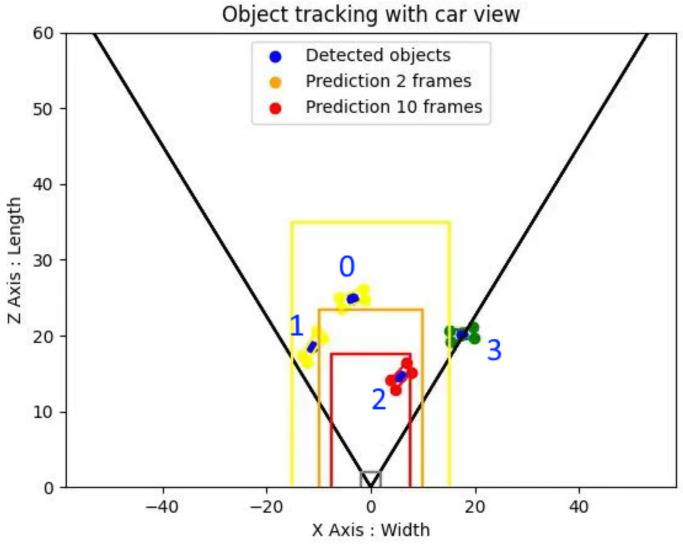
Visualization of 2D object tracking and predicted object trajectories. The numbers correspond to the tracking number of the detected vehicles, while their color corresponds to the position of the vehicles.

Additionally, in order to have better visibility on the trajectories of the surrounding objects, we realized a visualization tool, seen in Figure 2, that allows easy observation of the location and the trajectory of the tracked objects, with the libraries OpenCV [40] for image processing and Matplotlib [43] for graphic rendering.

Once the vehicles were represented on the 2D plane, we added the estimation of their trajectory, obtained through our modified version of SORT, to visualize whether there was any risk of collision between the vehicles and our own car. We then modeled three zones (yellow, orange, and red) to model different levels of danger, depending on the proximity to the obstacle with OpenCV. The color of the bounding boxes was associated with the danger zone for better clarity.

### 3.3. Dataset Combination

Combining and expanding the training dataset has a dual purpose. Firstly, increasing the quantity of the training data means increasing the diversity of represented situations, allowing the model to adapt to those newly represented situations. In addition, increased training data avoids over-fitting, and training coming from different datasets has the additional benefit of mitigating dataset-related bias, such as specific lighting conditions or scene over-representation.

The differences between the data provided by the KITTI and the GTA V datasets are shown in Table 3. As we can see, GTAV provided us with about 10 times the number of pictures provided by KITTI. As we had a majority of annotated data coming from our custom GTA V dataset, we adjusted KITTI’s data to follow GTA V’s data formatting, through resizing and cropping, using the OpenCV [40] library.

Since the original combined dataset had a large disproportion between both synthetic and real images, and the training dataset and the test dataset, we created another combined dataset with, this time, only 50% of synthetic data, in order to see whether the disproportion negatively affected the precision of the trained model or not. The new dataset combinations are shown in Table 4. All the datasets were used to train the large version of the YOLOv5-3d algorithm [1]. For additional results, we also comparef the 50% synthetic-real dataset with the KITTI dataset when both of them used a pre-training of 15 epochs on KITTI. Regardless of the dataset, in order to effectively compare them we needed to use the same validation test, which was extracted from KITTI.

## 4. Field Integration

**Figure 3:**
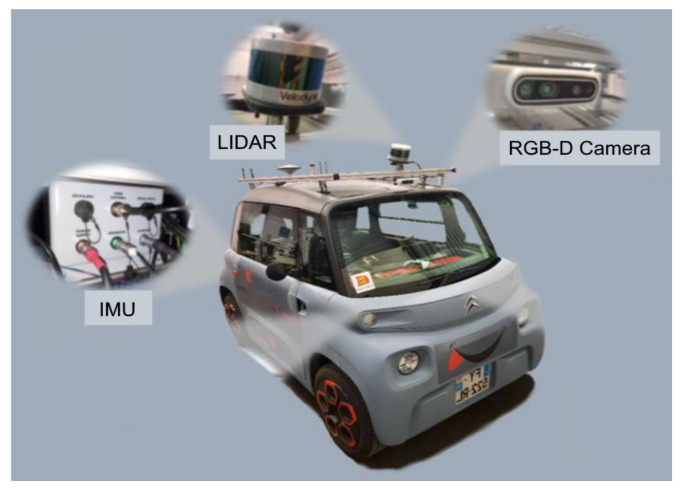
The Citroën AMI modified by the ESIGELEC for data recordings.

In order to carry out field testing and see how the algorithms behaved in real conditions, the next step of the work carried out was the integration of the modified Yolov5 algorithm on a road vehicle, in this case, a Citroën AMI (Figure 3) belonging to the ESIGELEC. We used the AMI vehicle, instrumented, and secured by ESIGELEC’s Innovation R&D department to carry out the real-time detection tests, equipped with both a Jetson TX2 and an RGB camera.

**Figure 4:**
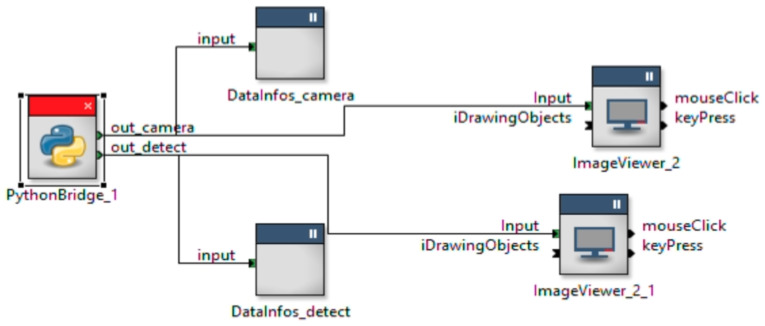
RTMaps diagram. We used the library “Python Bridge” of RTMaps in order to integrate the Yolov5 algorithm on the Jetson TX2.

To use the code on the Jetson, we used RTMaps (Figure 4, a run-time environment allowing users to record and replay data from vehicle sensors and buses. A connection is established between the Jetson and the laptop through TCP/IP because the embedded version of RTMaps requires it to be monitored by RTMaps studio.

## 5. Experimental Results

### 5.1. Dataset Combination

**Figure 5:**
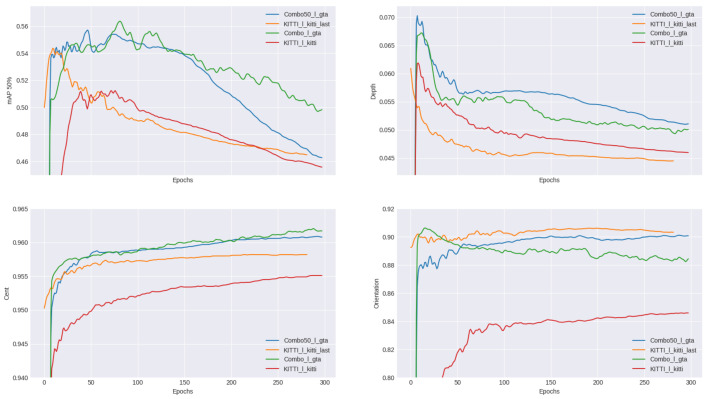
Inference results comparison using the previous models.

To see the effect of the dataset combination, we trained our model on the different combined and reference models described in Table 4. The results can be seen in Figure 5.

Here, we can see in green and blue the performance of the model trained on combined models (50% synthetic-real combination with pre-training in blue and regular combination in green), while in orange and red are the results with the original KITTI database (with pre-training in orange, no pre-training in red). We compared these models on 4 metrics: mAP (mean Average Precision), depth accuracy, center position accuracy, and, finally, orientation accuracy. The combined models allowed for increased performances in mAP, accuracy, and center position, with only the orientation not seeing any improvement. It was the only case in which the model trained on the KITTI dataset with pre-training performed slightly better than the models trained on the combined datasets. Nevertheless, given that the combination resulted in an mAP, center position, and depth accuracy improvement, we concluded that using synthetic data in addition to the real improves performances, despite the artificial nature of this data.

### 5.2. Knowledge Distillation

Knowledge distillation is an optimization method, as we have seen previously. We parallelized two networks, one in inference, the other in training, and we added a new type of loss function to the student network from the predictions made by the teacher network, allowing the student to learn from the ground truths and the teacher’s loss. We then proceeded with the training of the student model from the teacher on the CRIANN supercomputer.

**Figure 6:**
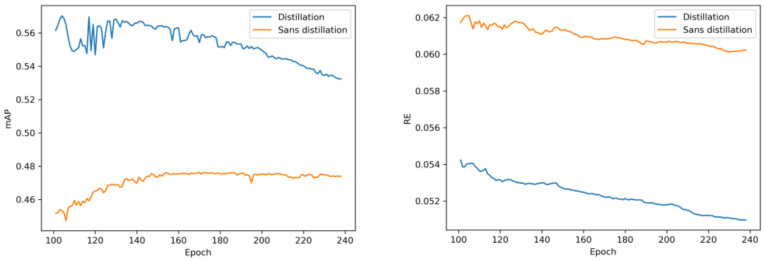
Comparison of the training on KITTI between a distilled and a classic model.

As can be seen in Table 5, our distillation model did not lead to increased real-time performance, but it nevertheless did allow for increased precision. As we can see in Figure 6, the model with distillation (in blue) achieved a higher mAP and a lower relative error, while maintaining the same real-time performance (Table 5). This is a valuable trade-off, as it enables us to improve the accuracy of our model without sacrificing speed. This is especially useful in applications where real-time performance is critical, such as in autonomous systems or high-speed data processing, where maintaining high accuracy, while keeping real-time performance constant, is essential.

### 5.3. Field Integration

**Figure 7:**
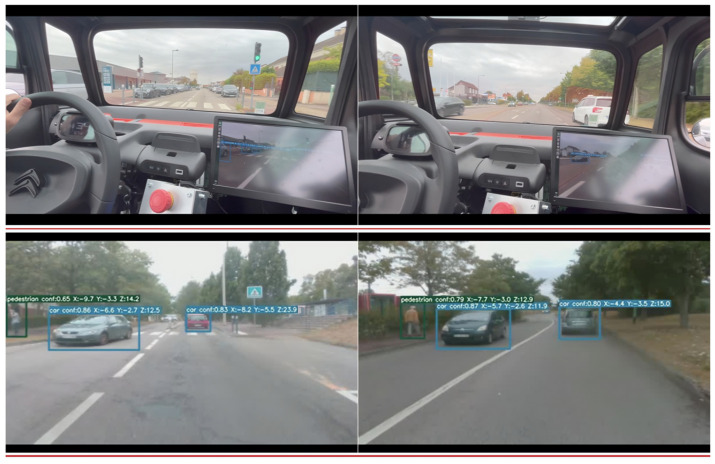
The algorithm tested in real-time conditions on an embedded Jetson.

Once all the modifications and installations on the road vehicle were done, we moved on to testing, in the urban areas of Rouen. We chose a trajectory that would allow us to meet a maximum of vehicles, people, and bicycles and also allowed us to drive up to 40 km/h; allowing us to test the algorithm in real working conditions, as seen in Figure 7.

### 5.4. Lightweight Model

We first trained YOLOv5 with the same dimensions as this new model on the COCO dataset for 300 epochs. The pre-trained weights of the first layers were then transferred to our model for 15 epochs of training on GTA. Finally, we trained this model on the KITTI and NuScenes datasets. This new model corresponded to the best compromise between low computational time and accuracy, as shown in Table 1.

The results from Table 1 are especially promising, because they allowed us to reach an inference time of 28 ms on the Jetson TX2 (corresponding to a frame-rate of 35 Frame-Per-Second), which is an embedded system with limited resources. In comparison to the light model, we went from an inference time of 70 ms to 28 ms (a 60% relative improvement), while only going from a mAP@0.5 value of 0.500 to 0.469 (a 6.2% decrease).

## 6. Conclusions

In conclusion, we were able to improve 3D object detection through the use of knowledge distillation, dataset combination, and the creation of a lightweight model. These techniques can effectively improve the performance of 3D object detection models without changes in the algorithms themselves and can be applied to different models. Combining multiple datasets allows the model to learn from a larger, more diverse set of data, which, in turn, improves its performance by exposing it to more situations it might encounter. The results of this study showed that combining multiple datasets improved the model’s performance without any drawbacks. The use of knowledge distillation allowed for the transfer of knowledge from a larger, pre-trained model to a smaller, more efficient model. In this work, while the use of knowledge distillation did not result in direct real-time performance improvement, it nevertheless resulted in improved accuracy of the small model. Overall, this work demonstrated the effectiveness of these techniques for improving the performance of 3D object detection models. Every approach separately provides some improvements to the model, whether in performance or in accuracy, and combining them and using them together is an essential step towards developing more efficient and effective 3D object detection systems for a variety of applications.

In this work, we focused on improving 3D object detection through the use of knowledge distillation, dataset combination, and the creation of a lightweight model. While these techniques were effective in improving the performance of our 3D object detection model, we believe that there are further improvements that can be made to our model. Firstly, we plan to implement vertical orientation prediction in our model. Currently, our model is only able to predict the horizontal orientation of objects. By adding vertical orientation prediction, we believe that our model will be able to better understand the 3D structure of the scene and will be more robust during use in field conditions. We are also considering changing the tracking method from SORT to a more modern method like DeepSORT [34] or Trackformer [35]. While these methods have shown improved accuracy compared to SORT, they are also more resource-intensive methods which would negatively affect the real-time performance. Finally, we plan to further use transfer learning methods to improve the performance of our model. We already had good results with transfer learning during this work, and we believe that using new methods would allow us to further improve the performance of our 3D object detection model. Overall, there is a lot of room for further improvement, as every aspect and technique used in this work is still evolving, and keeping up with the state-of-the-art allows us to keep improving our 3D detection models.

The article presents a novel approach to 3D object detection optimization by creating a lightweight model. Although this model has reduced accuracy, it has allowed for doubling the speed of the inference when used on the embedded Jetson TX2. Then, we adapted a 2D tracking method to the 3D field to increase the possible uses of the algorithm without loss of performance, which is the most important aspect of our approach. The article also discusses the knowledge distillation of a larger model into a smaller one, which did not provide performance gain as expected but did, however, provide accuracy gains without negative performance impact. The proposed method was implemented on an autonomous vehicle through an embedded system and field tested, demonstrating its potential practical applications in the field of road and railway smart mobility beyond theoretical use on high-end GPUs. Overall, the creation of lightweight models and knowledge distillation are both innovative approaches that show promise in improving real-time 3D object detection and tracking in embedded systems.

## Figures and Tables

**Table 1:** Quantitative results of our new optimal model on a Jetson TX2. The tested models were trained on KITTI and NuScenes.

Database (Model)	Resolution	2D Detection			Distance							Dimensions	Centre	Orientation	# Parameters	Time/img
		AP	R	mAP@0.5	RE	SRE	RMSE	log RMSE	$\alpha_{1}$	$\alpha_{2}$	$\alpha_{3}$	DS	CS	OS		
KITTI (Small)	672 × 224	0.722	0.520	0.500	0.069	0.163	2.27	0.094	0.978	0.997	0.999	0.863	0.942	0.879	7.3 M	70 ms
KITTI (LW)	608 × 192	0.642	0.518	0.469	0.056	0.133	2.15	0.081	0.985	0.998	0.999	0.871	0.953	0.863	6.0 M	28 ms
NuScenes (LW)	608 × 352	0.527	0.431	0.374	0.054	0.205	3.11	0.074	0.985	1	1	0.838	0.962	0.920	6.0 M	28 ms

**Table 2:** Quantitative evaluation of YOLOv5-3d on KITTI after training on different databases.

Training Database	Training Resolution	Inference Resolution	2D Detection			Distance							Dimension DS	Centre CS	Orientation OS
			AP	R	mAP@0.5	RE	SRE	RMSE	log RMSE	$\alpha_{1}$	$\alpha_{2}$	$\alpha_{3}$			
KITTI	672 × 244	672 × 244	0.897	0.752	0.732	0.0483	0.0987	1.82	0.0733	0.989	0.998	0.999	0.883	0.959	0.906
	672 × 244	1312 × 416	0.835	0.821	0.779	0.178	1.15	6.61	0.218	0.6	0.997	0.999	0.866	0.949	0.928
GTA	1312 × 768	1312 × 416	0.839	0.603	0.584	0.140	0.784	5.21	0.157	0.846	0.999	1.0	0.851	0.967	0.955
	1312 × 768	1312 × 768	0.823	0.593	0.571	0.114	0.584	4.63	0.134	0.913	1.0	1.0	0.848	0.962	0.955
NuScenes	1312 × 768	1312 × 416	0.857	0.678	0.697	0.455	5.97	14.7	0.378	0.0766	0.945	0.999	0.651	0.965	0.977
	1312 × 768	1312 × 768	0.843	0.642	0.635	0.0654	0.198	2.72	0.0776	0.994	1.0	1.0	0.654	0.964	0.986

**Table 3:** Data distribution between KITTI and our GTA dataset.

	Real Data	Synthetic Data	Combined Data
Dataset	KITTI	GTA V	KITTI + GTA V
N° of Pictures	11,193	110,271	121,464
Resolution	1224 × 370	1280 × 720	1280 × 720
N° of classes	3 (cars, pedestrians, cyclists)	3 (cars, pedestrians, cyclists)	3 (cars, pedestrians, cyclists)

**Table 4:** Comparison between the KITTI dataset and datasets combining both KITTI and synthetic data.

	Training Split	Validation Split (KITTI)	Hyper-Parameters	Model/Pre-Training
Combo	114,688	3769	GTA V	Large
Combo 50% synthetic	7424 synthetic + 7424 real	3769	GTA V	Large/last_15
KITTI (reference)	7424	3769	KITTI	Large
KITTI	7424	3769	KITTI	Large/large_15

**Table 5:** Comparison between a distilled model with a regular model, on the KITTI dataset, on a RTX 3080 GPU.

Method	GTA Pre-Training	Resolution	[val]			[val]			Time/img (ms)	Memory Consumption
			IOU 0.7			IOU 0.5				
			Easy	Mod	Hard	Easy	Mod	Hard		
Small		672 × 224	7.29	5.48	5.34	44.35	31.80	29.91	1.5	1.6 GB
		1312 × 416	15.52	13.27	13.19	48.24	36.14	31.78	4.4	1.7 GB
	X	1312 × 416	15.59	13.50	13.14	49.71	38.02	32.70	4.4	1.6 GB
Small (with distillation)	X	672 × 224	16.60	13.49	12.01	53.62	35.97	33.32	1.5	1.7 GB

## Data Availability

All the data used for training comes from KITTI [20], NuScenes [21], as well as data extracted from the video game GTA [28].

## Sample Availability

Samples of the compounds are available from the authors.

## Abbreviations

The following abbreviations are used in this manuscript:

CNN	Convolutional Neural Networks
COCO	Common Objects in Context
CRIANN	Centre Régional Informatique et d’Applications Numériques de Normandie
	(Regional Center for Computer Science and Digital Applications of Normandy)
ESRORAD	Esigelec engineering high school and Segula technologies ROad and RAilway Dataset
FPS	Frame-Per-Second
GPS	Global Positioning System
GTA	Grand Theft Auto
IMU	Inertial Measurement Unit
KITTI	Karlsruhe Institute of Technology & Toyota Technological Institute at Chicago
	vision benchmark suite
LIDAR	Light Detection And Ranging
mAP	mean Average Precision
MOT	Multi-Object Tracking
NUScenes	NuTonomy Scenes
SORT	Simple Online and Realtime Tracking
SOTA	State Of The Art
SYNTHIA	SYNTHetic Collection of Imagery and Annotations
YOLO	You Look Only Once
